# Development of renal renin-expressing cells does not involve PDGF-B-PDGFR-*β* signaling

**DOI:** 10.1002/phy2.132

**Published:** 2013-10-24

**Authors:** Bjoern Neubauer, Katharina Machura, Verena Rupp, Michelle D Tallquist, Christer Betsholtz, Maria Luisa S Sequeira-Lopez, R Ariel Gomez, Charlotte Wagner

**Affiliations:** 1 Institute of Physiology, University of RegensburgRegensburg, Germany; 2 Center for Cardiovascular Research, John A. Burns School of Medicine, University of HawaiiHonolulu; 3 Department of Medical Biochemistry and Biophysics, Karolinska Institutet StockholmStockholm, Sweden; 4 Department of Pediatrics, University of Viriginia School of MedicineCharlottesville

**Keywords:** Mural cells, PDGF-B, PDGF-*β* receptor, pericyte, renin

## Abstract

Apart from their endocrine functions renin-expressing cells play an important functional role as mural cells of the developing preglomerular arteriolar vessel tree in the kidney. The recruitment of renin-expressing cells from the mesenchyme to the vessel wall is not well understood. Assuming that it may follow more general lines of pericyte recruitment to endothelial tubes we have now investigated the relevance of the platelet-derived growth factor (PDGF)-B-PDGFR-*β* signaling pathway in this context. We studied renin expression in kidneys lacking PDGFR-*β* in these cells and in kidneys with reduced endothelial PDGF-B expression. We found that expression of renin in the kidneys under normal and stimulated conditions was not different from wild-type kidneys. As expected, PDGFR-*β* immunoreactivity was found in mesangial, adventitial and tubulo-interstitial cells but not in renin-expressing cells. These findings suggest that the PDGF-B-PDGFR-*β* signaling pathway is not essential for the recruitment of renin-expressing cells to preglomerular vessel walls in the kidney.

## Introduction

Renin-producing cells in the kidney fulfill multiple functions. Apart from their endocrine function as key regulators of the activity of the renin angiotensin system (RAS), renin-producing cells also form the mural layers of the developing preglomerular vascular tree (Sauter et al. 2008) and potentially regulate angiogenesis in the developing kidney (Reddi et al. 1998). With ongoing kidney maturation renin-expressing cells reversibly transform into vascular smooth muscle-like cells. In the adult kidney renin-producing cells form mural cells only at the glomerular ends of afferent arterioles, leading to the characteristic impression of juxtaglomerular renin expression. First, renin-positive cells within the renal mesenchyme become visible at embryonic day 14 in the mouse kidney (Sequeira Lopez et al. 2001). It is still an open question how renin-expressing cells are attracted to developing vessels to stabilize the vessel wall by forming mural cell layers. Commonly, mural cells are attracted to endothelial tubes in the form of pericytes (Gerhardt and Betsholtz 2003; von Tell et al. 2006; Senger and Davis 2011; Stratman and Davis 2012). The interaction between endothelial cells and pericytes is a very active field of research (Gerhardt and Betsholtz 2003; Stratman and Davis 2012). It is thought that both cell types communicate via soluble paracrine and via membrane-bound factors. An important signaling pathway of membrane-bound factors comprises the Notch-signaling pathway (McCright et al. 2001; Alva and Iruela-Arispe 2004). In fact, recent studies have confirmed that the Notch-signaling pathway is also very important for activation and maintenance of renin cells in the kidney. The intracellular domain of Notch1 has been shown to activate the renin promoter (Pan et al. 2005) and the deletion of the RBP-J transcription factor which is the common downstream effector of all Notch-receptors from the renin cell lineage leads to altered preglomerular vessels with thin cells. Renin-producing cells along the vessels are strongly reduced in number. Moreover, the typical reactivation of vascular renin expression in the adult kidney induced by chronic challenges of the RAS is absent if RBP-J is deleted from the renin cell lineage (Castellanos Rivera et al. 2011).

Another well-characterized signaling pathway relevant to the recruitment of pericytes to endothelial tubes comprises platelet-derived growth factor signaling. Endothelium-derived PDGF-B binds to PDGF receptor-*β* on pericytes (Hellstrom et al. 1999; Gerhardt and Betsholtz 2003; Abramsson et al. 2007), which represents a receptor tyrosine kinase. After dimerization several signal transduction pathways can be induced, including the MAP kinase and the PI3 kinase pathways (Hoch and Soriano 2003). For the reason that PDGF signaling is known to control migration and adhesion of mural cells and that renin-producing cells represent a component of the arterial wall, we tested for the possibility that renin-producing cells might also be recruited to endothelial tubes by this pathway. In addition, our assumption is based on the observation that renin-producing cells just as renal vascular smooth muscle cells and mesangial cells derive from Foxd1-positive cells of the renal mesenchyme (Sequeira Lopez and Gomez 2011). In this context, studies on PDGF-B and PDGFR-*β* knockout (KO) mice showed that they did not develop mesangial cells and had also defects in vessel wall formation (Leveen et al. 1994; Soriano 1994; Lindahl et al. 1997; Hellstrom et al. 1999, 2001; Ohlsson et al. 1999). Thus, we studied the distribution of renin-expressing cells in kidneys in which the PDGFR-*β* had been deleted from renin-expressing cells and their descendants to find out whether the recruitment of these cells from the renal mesenchyme to the vessel wall follows the same signaling pathway as the attraction of vascular smooth muscle cells and mesangial cells. In consideration of the possibility that the renin gene might be induced rather late during kidney development, namely after the adhesion of the cells to the vessel wall, we used a second approach in which endothelial expression of PDGF-B was reduced.

## Methods

### Animals

Animal experiments were conducted according to the National Institutes of Health guidelines for the care and use of animals in research and were approved by the local ethics committee.

**Figure 1:**
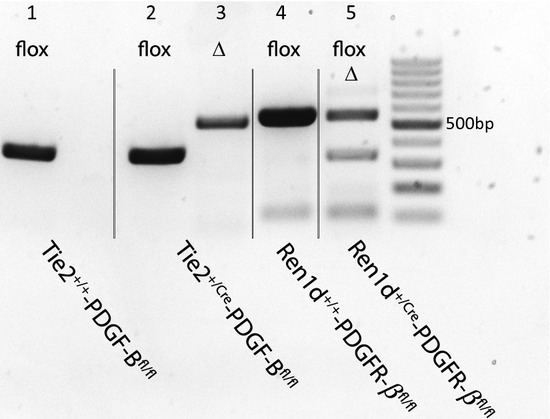
In vivo recombination of PDGF-B and PDGFR-*β* shown by PCR genotyping of renal cortex preparations. Δ-fragments represent Cre-recombinase-mediated deletion of PDGF-B (lane 3; 540 bp) or PDGFR-*β* (lane 5; 350 bp) of floxed PDGF-B (lanes 1, 2; 400 bp) or PDGFR-*β* (lanes 4, 5; 520 bp) genes.

Mice with a floxed PDGF-B gene or PDGFR-*β* receptor gene were generated by Enge and collegues (2002) and Schmahl et al. (2008), respectively. Renin-Cre (Ren1d^+/Cre^) (Sequeira Lopez et al. 2004) and Tie-2-Cre (Tie-2^+/Cre^) (Kisanuki et al. 2001) mice were used as described previously by Wagner et al. (2010). Mice were mated to generate offspring being homozygous for the floxed PDGF-B (PDGF-B^fl/fl^) or PDGFR-*β* gene (PDGFR-*β*^fl/fl^) carrying Tie2-Cre or Renin-Cre. Corresponding littermates exhibiting no Cre-recombinase (Tie-2^+/+^-PDGF-B^fl/fl^, Ren1d^+/+^-PDGFR-*β*^fl/fl^) served as controls. Genotyping was performed by polymerase chain reaction (PCR) from tail-tip biopsies for detection of Ren1d^+/Cre^ and Tie-2^+/Cre^ as described previously (Wagner et al. 2010). For the floxed PDGF-B and PDGFR-*β* gene the following primers were used: PDGF-B: BF: 5′-GGGTGGGACTTTGGTGTAGAGAAG-3′; BB1: 5′-TTTGAAGCGTGCAGAATGCC-3′; BB2: 5′-GGAACGGATTTTGGAGGTAGTGTC-3′; BBlox:-5′-TCTGGGTCACTGCTTCAGAATAGC-3′ (Enge et al. 2002); PDGFR-*β*: I: 5′-GGAAAAGCAGGTTTGTGC-3′; II: 5′-TACCAGGAAGGCTTGGGAAG-3′; III: 5′-CCAGTTAGTCCACTTATGTTG-3′ (Schmahl et al. 2008). In vivo recombination of PDGF-B and PDGFR-*β* was verified by PCR analysis of renal cortex preparations of adult mice or whole kidneys (E18, pp1) as described by Enge et al. (2002) and Schmahl et al. (2008) (Fig. 1).

### Sample collection

Male 8–16 weeks old Tie-2^+/Cre^-PDGF-B^fl/fl^ mice, Ren1d^+/Cre^-PDGFR-*β*^fl/fl^ mice and their corresponding controls were split into two groups with *n* ≥ 6. One group of each was kept under standard conditions (NaCl 0.6% *w/w*), the other group on low-salt diet (NaCl 0.02% *w/w*) for 10 days. For the last 5 days of the diet, these animals additionally received the angiotensin I – converting enzyme (ACE) inhibitor enalapril (10 mg/kg × day) via drinking water. After treatment, the animals were deeply anesthetized with isoflurane and one kidney was snap frozen in liquid nitrogen for RNA analysis. The other kidney was fixed in situ by retrograde perfusion with 3% paraformaldehyde (PFA) for 3D-reconstruction and immunohistochemical analysis. Kidneys of E18 were collected from fetuses 18 days after detection of vaginal plugs or 1 day after birth (pp1). Pups were split equally and kidneys were either perfusion-fixed by left-ventricular cannulation or snap frozen for mRNA isolation.

### Three dimensional (3-D) reconstruction

For three dimensional reconstruction of arterial trees, serial slices (5 μm) of paraffin embedded kidneys were immunofluorescently stained against *α*-smooth muscle actin (*α*-SMA) and renin, digitized, and reconstructed by the aid of AMIRA software (FEI, Berlin, Germany). The method of 3-D reconstruction and analysis is described in detail by Sauter et al. (2008).

### Immunohistochemistry

Perfusion-fixed (3% PFA in phosphate-buffered saline) kidneys were dehydrated by a graded series of alcohol solutions (70, 80, 90, and 100% methanol 2× in each), followed by two times in 100% isopropanol for 0.5 h each and embedded in paraffin.

Immunolabeling was performed on 5-μm paraffin sections. After dewaxing (in 100% xylol) and rehydrating (graded series of isopropanol 100%-70%-water), the tissue slices were immunostained against the specified antigens.

Tissues were blocked with 10% horse serum and 1% bovine serum albumin in PBS for 0.5 h at room temperature. The sections then were incubated overnight at 4°C with the respective primary antibodies. After several washes, tissues were incubated with secondary antibodies for 90 min at room temperature. The following antibodies were used: Chicken anti-renin IgG (diluted 1:400; Davids Biotechnologie, Regensburg, Germany); mouse anti-*α*-SMA IgG (diluted 1:600; Abcam, Cambridge, U.K.); rabbit anti-PDGFR-*β* IgG (diluted 1:200; Abcam); Cy2-conjugated donkey anti-chicken IgG; rhodamine (TRITC)-conjugated donkey anti-mouse IgG; rhodamine (TRITC)-conjugated donkey anti-rabbit IgG; Cy5-conjugated donkey anti-mouse IgG. All secondary antibodies were purchased from Dianova (Hamburg, Germany) and diluted 1:400. All sections for fluorescent imaging were mounted with glycergel (Dako, Hamburg, Germany). Based on *α*-SMA staining the wall thickness of afferent arterioles was measured in micrographs at a magnification of 400× using AxioVision LE 4.5 software (Zeiss, Göttingen, Germany). Analyses of 33–51 individual vessels of 4–5 kidneys per genotype were performed.

### mRNA determination

Total RNA was isolated from frozen kidneys and reverse transcribed into cDNA. Real-time PCR measurements were performed in a Light Cycler 480 (Roche, Mannheim, Germany) using the provided LightCycler DNA Master SYBR Green I kit from Roche Molecular Biochemicals. The relevant primers and amplification protocols to analyze the mRNA expression were previously described by Machura et al. (2009) for GAPDH and renin mRNA.

### Blood pressure measurement

Determination of systolic blood pressure of conscious mice using a tail-cuff manometry device (TSE Systems, Bad Homburg, Germany) was described in detail before (Machura et al. 2013). Values are repeated measurements of 6 mice per genotype on 10 consecutive days.

### Statistical analysis

Values are provided as means ± SE. Differences between experimental groups were analyzed by analysis of variance (ANOVA) and Bonferroni's adjustment for multiple comparisons with *P* values less than 0.05 were considered as statistically significant.

## Results

**Figure 2:**
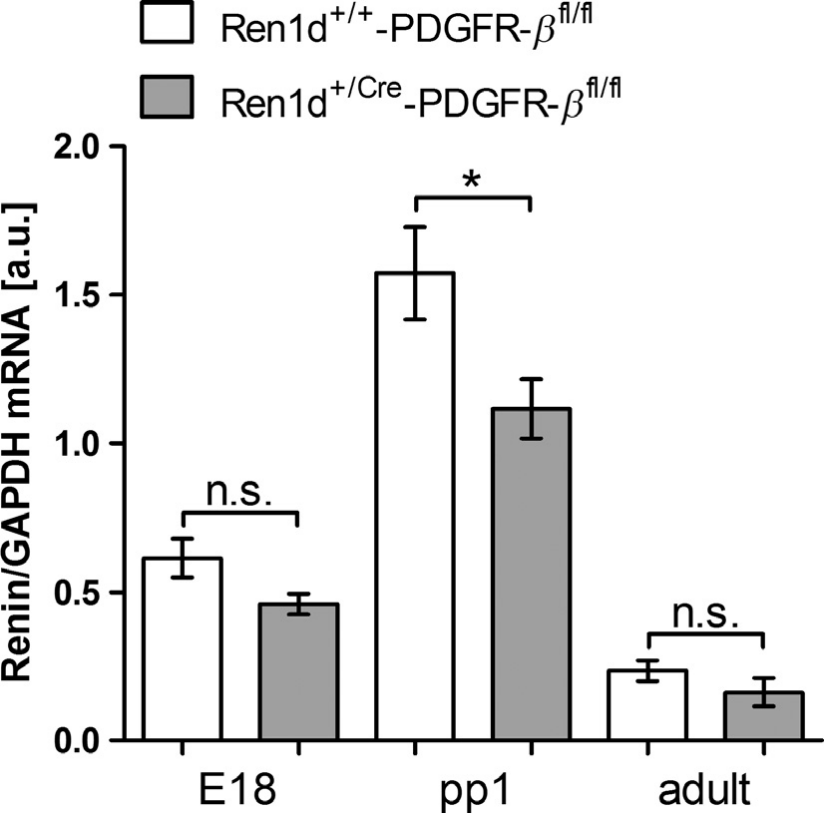
Renal renin mRNA levels during renal development in Ren1d^+/+^-PDGFR-*β*^fl/fl^ and Ren1d^+/Cre^-PDGFR-*β*^fl/fl^ mice at E18, pp1, and in adults. Data are means ± SE of 6–13 kidneys per developmental stage. E18, embryonic day 18; pp1, postpartum day 1; adult (8 weeks). *P = 0.028; n.s., not significant.

**Figure 3:**
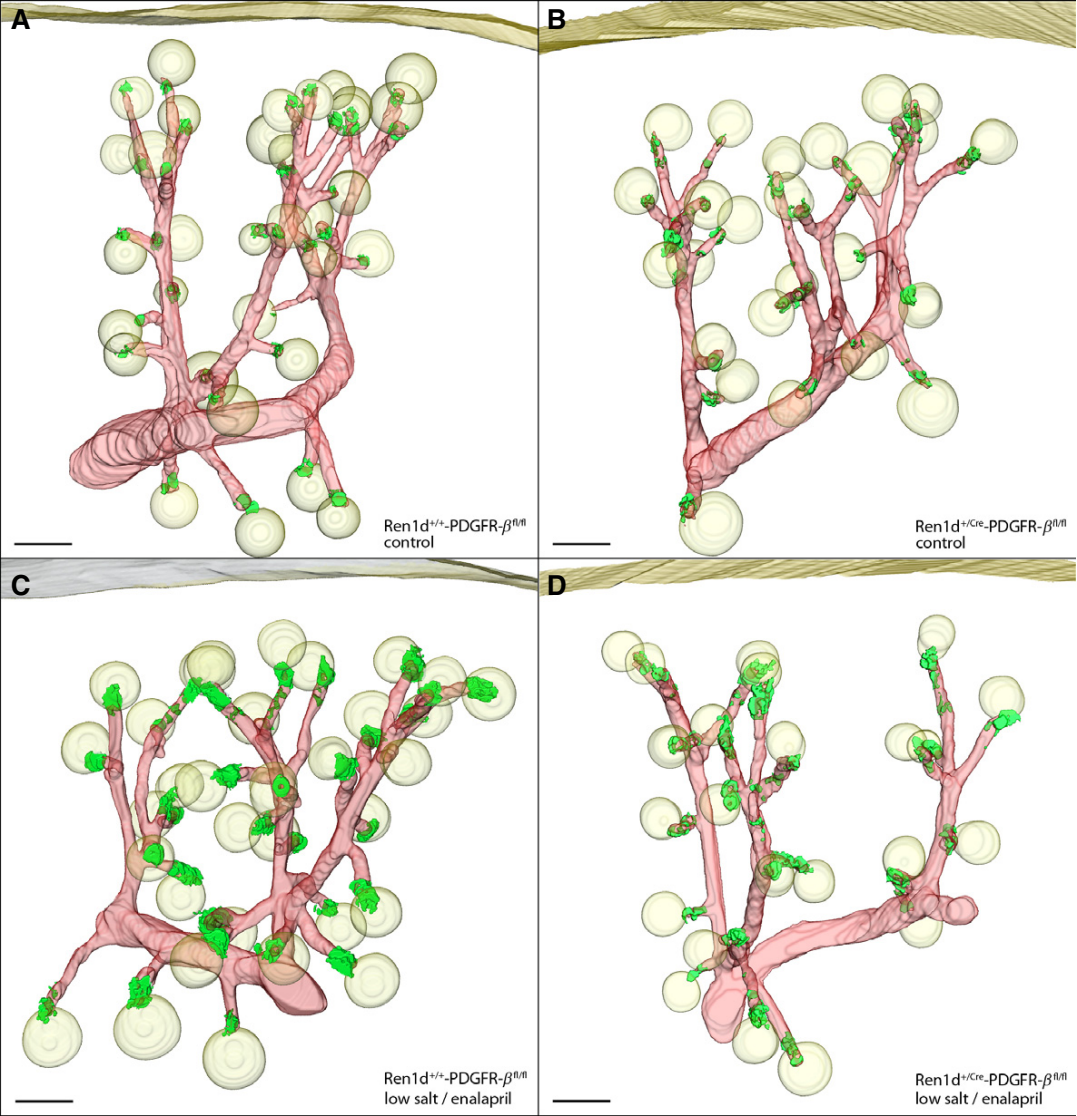
Renin expression in adult kidneys with conditional PDGFR-*β* deletion. 3D-Reconstruction of *α*-smooth muscle actin (*α*-SMA) immunoreactive vascular structures (red) and renin-immunoreactive areas (green) in kidneys of Ren1d^+/+^-PDGFR-*β*^fl/fl^ and Ren1d^+/Cre^-PDGFR-*β*^fl/fl^ mice under control conditions (A, B) and after treatment with low salt and enalapril (C, D). Glomeruli are displayed as yellow balls. Scale bar = 100 μm.

**Figure 4:**
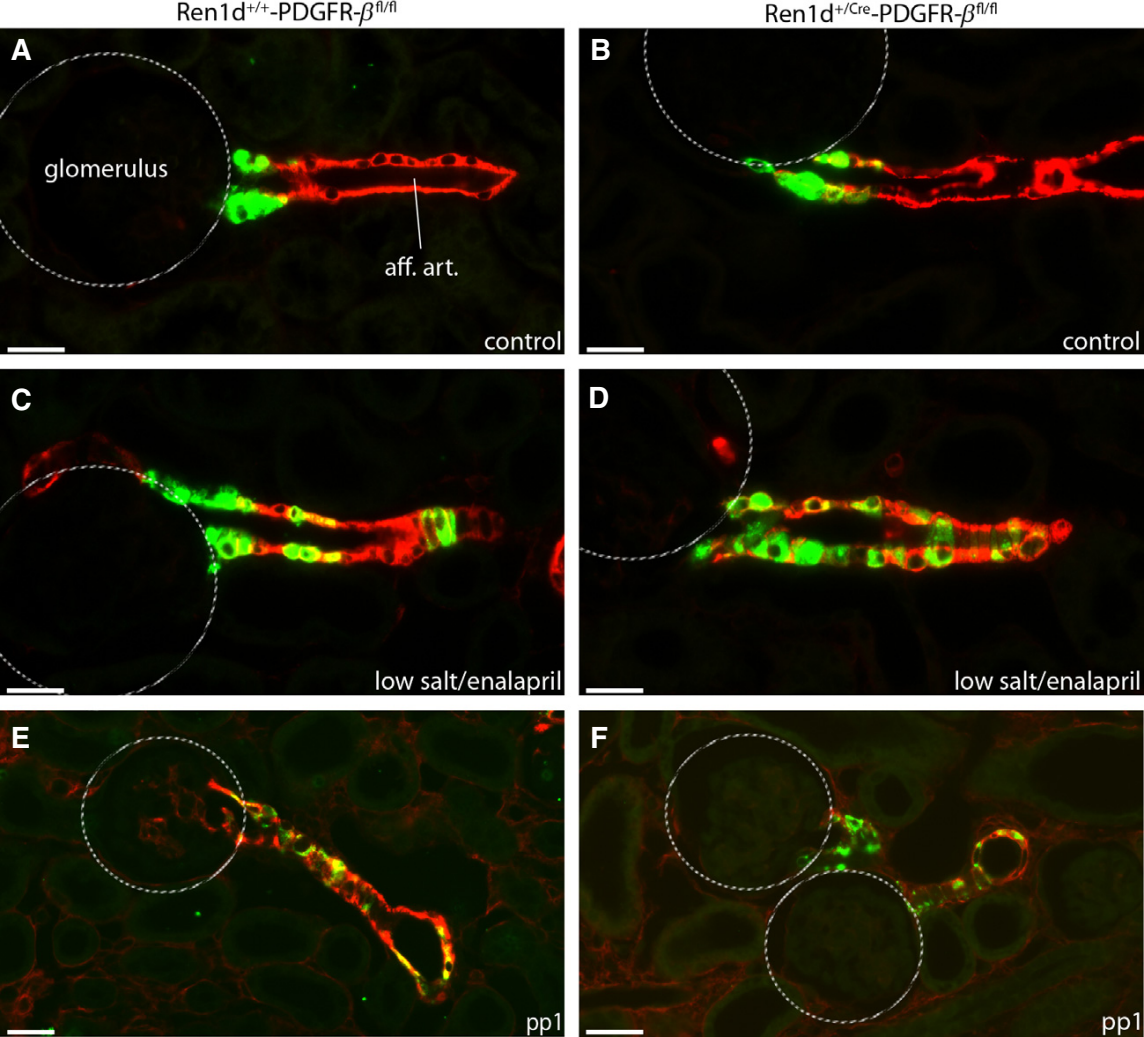
Immunohistochemical localization of renin-producing cells in Ren1d^+/+^-PDGFR-*β*^fl/fl^ and Ren1d^+/Cre^-PDGFR-*β*^fl/fl^ mice. Renin (green) and *α*-SMA (red) immunoreactivity in adult kidney sections under control conditions (A. B), after treatment with low salt and enalapril (C, D) and at developmental stage pp1 (E, F). Yellow color indicates coexpression. Scale bar = 20 μm.

**Figure 5:**
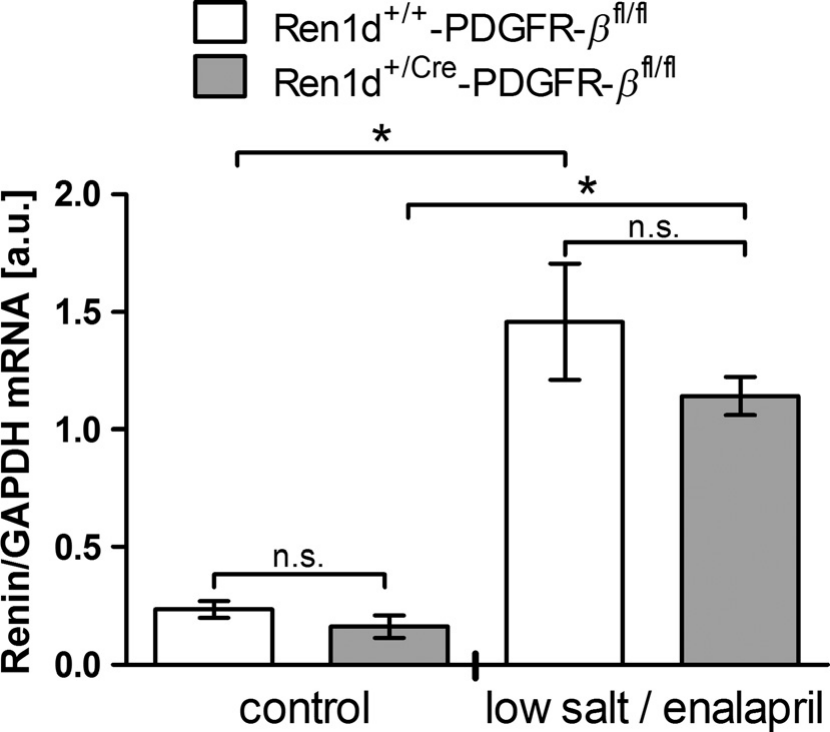
Renin mRNA expression in Ren1d^+/+^-PDGFR-*β*^fl/fl^ and Ren1d^+/Cre^-PDGFR-*β*^fl/fl^ mice under control conditions or after low-salt diet combined with enalapril. Data are means ± SE, 6–8 animals per group. *P < 0.05; n.s., not significant.

We first examined, whether deletion of the PDGFR-*β* receptor from renin-expressing cells and their descendants has influence on overall renin expression during kidney development. Therefore, renin mRNA abundance in kidneys of embryonic day 18 (E18), day 1 after birth (pp1) and of 8 weeks (adult) was determined in mice lacking PDGFR-*β* (genotype Ren1d^+/Cre^-PDGFR-*β*^fl/fl^) and compared to controls (genotype Ren1d^+/+^-PDGFR-*β*^fl/fl^). As shown in Figure 2**,** renin mRNA levels were highest at day 1 after birth. Although there was no marked difference of renin mRNA abundance between the two genotypes at E18 and in adults, however, a minor decrease in kidneys of Ren1d^+/Cre^-PDGFR-*β*^fl/fl^ mice versus controls was observed at pp1. We then looked for the overall distribution of renin expression in adult kidneys. 3D-reconstructions of the preglomerular vascular tree showed no obvious differences between the two genotypes (Fig. 3). Renin-expressing cells were located in typical juxtaglomerular position in the media layer of afferent arterioles (Fig. 4). Recruitment of renin cells during chronic challenge of the renin angiotensin system was examined by treating mice with a combination of low-salt diet and the ACE-inhibitor, enalapril. This treatment increased renin mRNA abundance five- to sixfold (Fig. 5) due to reexpression of renin in the media layer of afferent arterioles. Also under stimulating conditions there was no difference of renin expression between the two genotypes neither with regard to extent nor localization (Fig. 5). During the perinatal stage of renal development renin expression was mainly found in larger, for example, interlobular arteries and in afferent arterioles in a striped, discontinuous pattern. Again the developmental renin expression pattern in the conditional PDGFR-*β* KOs did not differ from their WT controls (Fig. 4 E–F).

**Figure 6:**
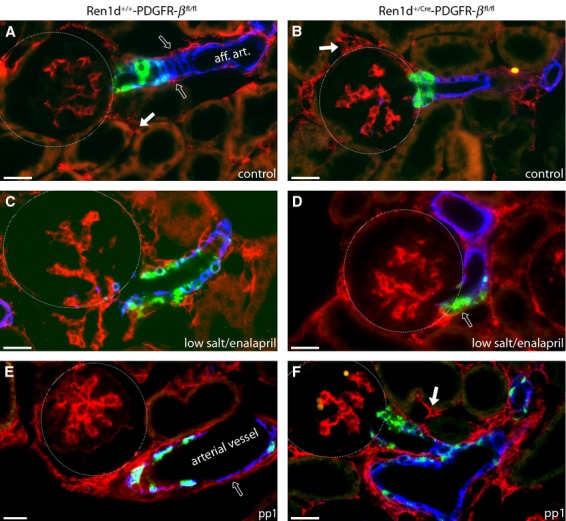
Localization of PDGF-*β* receptor in the kidney of adult and perinatal mice. Renin (green), *α*-SMA (blue), and PDGFR-*β* (red) immunoreactivity in kidney sections from Ren1d^+/+^-PDGFR-*β*^fl/fl^ and Ren1d^+/Cre^-PDGFR-*β*^fl/fl^ mice in adult kidneys under control conditions (A, B), after treatment with low salt and enalapril (C, D) and at developmental stage pp1 (E, F). Open arrows indicate PDGFR-*β* immunoreactivity in adventitial cells, closed arrows point to tubulo-interstitial cells. Turquoise color indicates coexpression of renin and *α*-SMA. Scale bar: 20 μm.

To check the efficiency of PDGFR-*β* deletion by the Cre-Lox system, we looked for PDGFR-*β* immunoreactivity in kidneys of the two genotypes (Fig. 6). However, there was no apparent difference of PDGFR-*β* immunoreactivity. PDGFR-*β* was well detectable in the glomerular mesangium, tubulo-interstitial cells and adventitial cells, but not in renin-expressing cells within the vessel walls, neither under basal nor stimulated conditions of adult kidneys nor in the perinatal period (Fig. 6). We also observed no difference between the genotypes regarding body weight nor blood pressure (data not shown).

**Figure 7:**
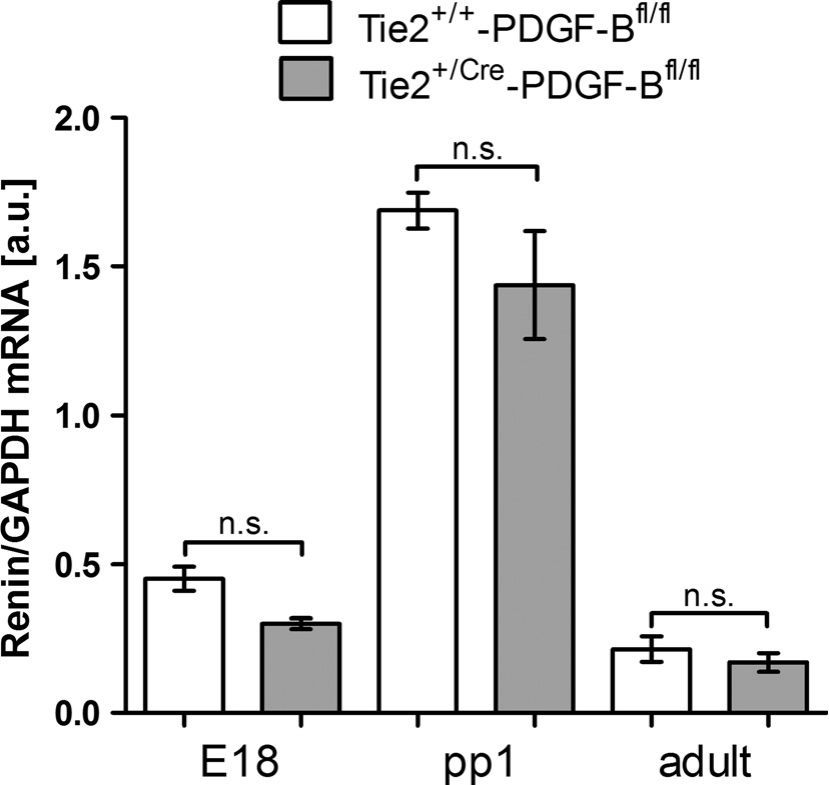
Renal renin mRNA levels during renal development in Tie2^+/+^-PDGF-B^fl/fl^ and Tie2^+/Cre^-PDGF-B^fl/fl^ mice at E18, pp1 and in adults. Data are means ± SE of 6–8 kidneys per developmental stage. E18, embryonic day 18; pp1, postpartum day 1; adult (8 weeks). n.s., not significant.

**Figure 8:**
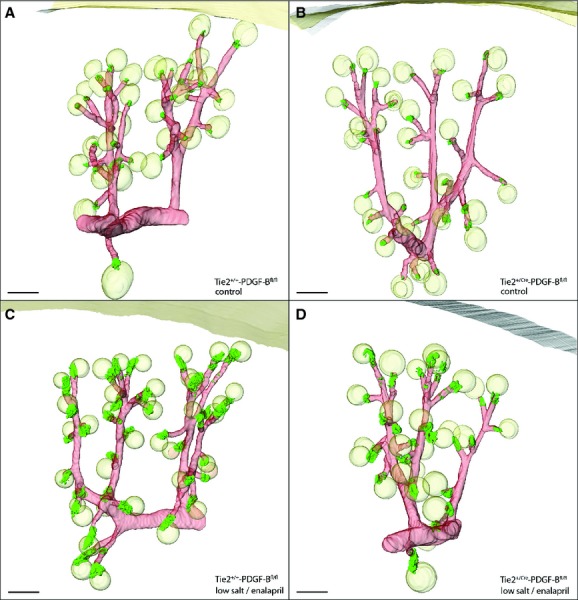
Renin expression in adult kidneys with conditional PDGF-B deletion. 3D-Reconstruction of *α*-SMA immunoreactive vascular structures (red) and renin-immunoreactive areas (green) in kidneys of Tie2^+/+^-PDGF-B^fl/fl^ and Tie2^+/Cre^-PDGF-B^fl/fl^ mice under control conditions (A, B) and during treatment with low salt and enalapril (C, D). Yellow balls represent glomeruli. Scale bar = 100 μm.

**Figure 9:**
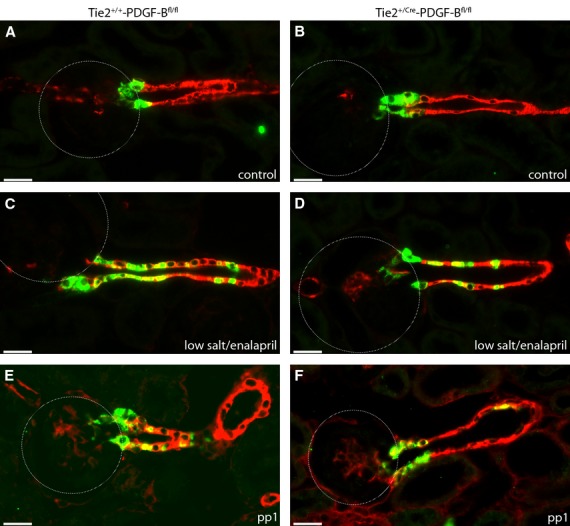
Immunohistochemical localization of renin-producing cells in Tie2^+/+^-PDGF-B^fl/fl^ and Tie2^+/Cre^-PDGF-B^fl/fl^ mice. Renin (green) and *α*-SMA (red) immunoreactivity in kidney sections of adult kidneys under control conditions (A, B), after treatment with low salt and enalapril (C, D) and at developmental stage pp1 (E, F). Yellow color indicates coexpression. Scale bar = 20 μm.

**Figure 10:**
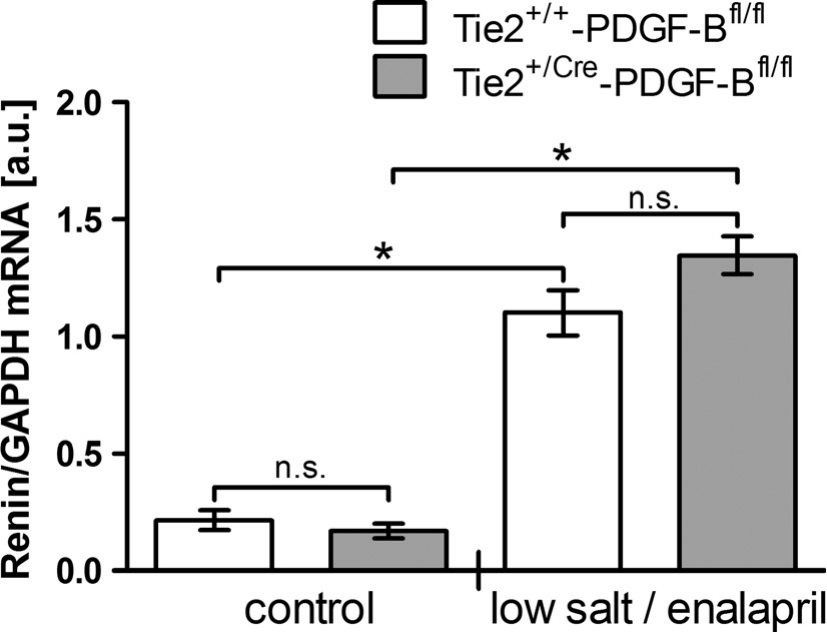
Renin mRNA expression in Tie2^+/+^-PDGF-B^fl/fl^ and Tie2^+/Cre^-PDGF-B^fl/fl^ mice under control conditions or after low-salt diet combined with enalapril. Data are means ± SE, 6 animals per group. *P < 0.05; n.s., not significant.

With regard to the possibility that the induction of the renin gene might happen at a point in time, at which the PDGF-B-PDGFR-*β* signaling is not any longer relevant for the recruitment, we aimed to target the recruitment of potential renin cell precursors to the vessel walls. Therefore, endothelial PDGF-B production was knocked down in mice (Tie2^+/Cre^-PDGF-B^fl/fl^) and the phenotype was compared with respective controls (Tie2^+/+^-PDGF-B^fl/fl^). Analogous to the experiments described above we first determined the developmental changes of renin mRNA abundances (Fig. 7). Compared to wild types (WT), renin mRNA levels were found to be unaltered in the kidneys of endothelium-specific PDGF-B KO mice at all developmental stages. Also systolic blood pressure of conscious mice was comparable (KO: 126.3 ± 2.36 mmHg (*N* = 59) vs. WT: 132.4 ± 2.20 (*N* = 59), *P* = 0.0583). We then looked for the overall distribution of renin expression in adult kidneys. 3D-reconstructions of the preglomerular vessel tree showed no obvious differences between the two genotypes with regard to the gross branching pattern and to the position of renin-expressing cells (Fig. 8). Renin expression was found in the media layer of afferent arterioles in juxtaglomerular position (Fig. 9). Chronic stimulation by treatment with a combination of low-salt diet and the ACE-inhibitor enalapril led to a significant increase in renin mRNA abundance (Fig. 10) and the typical upstream reexpression of renin in the media layer of afferent arterioles (Fig. 9). Also under stimulating conditions there was no difference between renin expression of the two genotypes with regard to neither extent nor localization (Fig. 10). At postpartum day 1 the conditional PDGF-*β* KO showed a somewhat reduced wall thickness of the afferent arterioles (3.15 ± 0.12 μm [*N* = 33] vs. 4.52 ± 0.19 μm [*N* = 51], *P* < 0.001) in respect of *α*-SMA-immunohistochemistry, but renin-expressing cells were normal in distribution and in quantity (Fig. 9E–F).

## Discussion

The aim of this study was to further elucidate the mechanisms that recruit renin-producing cells to preglomerular vessels in the kidney. After the recent demonstration of the relevance of the Notch pathway in this context (Castellanos Rivera et al. 2011) we now considered the relevance of the PDGF-B-PDGFR-*β* signaling pathway, which appears to be of similar relevance for the recruitment of pericytes to renal vessel walls as the Notch pathway (McCright et al. 2001; Bjarnegard et al. 2004). For this purpose we studied the role of PDGFR-*β* signaling in the development and function of the renin cell lineage. Deletion of PDGFR-*β* had no consequence on renin location during kidney development nor in the adult kidney. The reduced renin expression, which was observed at pp1 is most likely due to less precise assessment of the time of fertilization, as we know from previous studies, that the abundance of renin mRNA is profoundly time dependent around the time of birth (Machura et al. 2009; Neubauer et al. 2011). Apart from that renin expression was unaffected, what is supported by our observation that renin-immunoreactive cells lack PDGFR-*β*-immunoreactivity both during kidney development and in the adult kidney. One possibility was that renin expression is a rather late process that occurs after incorporation of the cells to the vessel wall and that PDGFR-*β* is already downregulated when renin expression is induced. Therefore, PDGFR-*β* would be dispensable at this stage.

To test for relevance of the PDGF-B-PDGFR-*β* signaling system for the recruitment of potential renin cell precursors, we knocked down endothelial PDGF-B production. Also this maneuver was without impact on the development and localization of renin-expressing cells during development and in the adult kidney. We cannot definitively exclude the fact that this lack of effect of endothelial PDGF-B deletion was due to potential PDGF-B signaling from adventitial cells (Meliss et al. 1999) or an insufficient inhibition of endothelial PDGF-B production (Enge et al. 2002). From experience with other floxed genes we and others learned that Tie-2 is a strong endothelial Cre driver (Wagner et al. 2010). The genotyping gel in fact confirmed effective Cre-lox recombination in the kidney (Fig. 1). A previous study using Tie-1-Cre to delete PDGF-B from endothelial cells, also reported no major abnormalities in adult kidneys (Bjarnegard et al. 2004). Only during the perinatal period glomeruli showed a striking “ballooning” due to the lack of mesangial cells (Bjarnegard et al. 2004). As we observed a similar phenomenon in this study using Tie-2-Cre, we assume an effective inhibition of endothelial PDGF-B production. As a consequence also this line of experiments does not support a relevant role of the PDGF-B-PDGFR-*β* signaling pathway for the recruitment of renin-expressing cells or their precursors to preglomerular vessel walls. This conclusion is further supported by data on renin expression in kidneys of neonatal PDGF-B deficient mice (Hellstrom et al. 1999). Also, in these mice renin-expressing cells were found in afferent arterioles and in juxtaglomerular position as in WT mice (Hellstrom et al. 1999). Thus, a clear difference between the recruitment of renin-expressing cells and typical mural cells to vascular walls appears to exist. As renin expression has been shown to be present in renal interstitial cells, especially during metanephrogenesis (Berg et al. 2013), renin-producing cells might represent a mesenchyme-derived cell type (Sequeira Lopez and Gomez 2011) with an attraction and integration pathway different from PDGF-B-PDGFR-*β* signaling.

## References

[b1] Abramsson A, Kurup S, Busse M, Yamada S, Lindblom P, Schallmeiner E (2007). Defective N-sulfation of heparan sulfate proteoglycans limits PDGF-BB binding and pericyte recruitment in vascular development. Genes Dev.

[b2] Alva JA, Iruela-Arispe ML (2004). Notch signaling in vascular morphogenesis. Curr. Opin. Hematol.

[b3] Berg AC, Chernavvsky-Sequeira C, Lindsey J, Gomez RA, Sequeira-Lopez ML (2013). Pericytes synthesize renin. World J. Nephrol.

[b4] Bjarnegard M, Enge M, Norlin J, Gustafsdottir S, Fredriksson S, Abramsson A (2004). Endothelium-specific ablation of PDGFB leads to pericyte loss and glomerular, cardiac and placental abnormalities. Development.

[b5] Castellanos Rivera RM, Monteagudo MC, Pentz ES, Glenn ST, Gross KW, Carretero O (2011). Transcriptional regulator RBP-J regulates the number and plasticity of renin cells. Physiol. Genomics.

[b6] Enge M, Bjarnegard M, Gerhardt H, Gustafsson E, Kalen M, Asker N (2002). Endothelium-specific platelet-derived growth factor-B ablation mimics diabetic retinopathy. EMBO J.

[b7] Gerhardt H, Betsholtz C (2003). Endothelial-pericyte interactions in angiogenesis. Cell Tissue Res.

[b8] Hellstrom M, Kalen M, Lindahl P, Abramsson A, Betsholtz C (1999). Role of PDGF-B and PDGFR-beta in recruitment of vascular smooth muscle cells and pericytes during embryonic blood vessel formation in the mouse. Development.

[b9] Hellstrom M, Gerhardt H, Kalen M, Li X, Eriksson U, Wolburg H (2001). Lack of pericytes leads to endothelial hyperplasia and abnormal vascular morphogenesis. J. Cell Biol.

[b10] Hoch RV, Soriano P (2003). Roles of PDGF in animal development. Development.

[b11] Kisanuki YY, Hammer RE, Miyazaki J, Williams SC, Richardson JA, Yanagisawa M (2001). Tie2-Cre transgenic mice: a new model for endothelial cell-lineage analysis in vivo. Dev. Biol.

[b12] Leveen P, Pekny M, Gebre-Medhin S, Swolin B, Larsson E, Betsholtz C (1994). Mice deficient for PDGF B show renal, cardiovascular, and hematological abnormalities. Genes Dev.

[b13] Lindahl P, Johansson BR, Leveen P, Betsholtz C (1997). Pericyte loss and microaneurysm formation in PDGF-B-deficient mice. Science.

[b14] Machura K, Steppan D, Neubauer B, Alenina N, Coffman TM, Facemire CS (2009). Developmental renin expression in mice with a defective renin-angiotensin system. Am. J. Physiol. Renal Physiol.

[b15] Machura K, Iankilevitch E, Neubauer B, Theuring F, Kurtz A (2013). The aldo-keto reductase AKR1B7 coexpresses with renin without influencing renin production and secretion. Am. J. Physiol. Renal. Physiol.

[b16] McCright B, Gao X, Shen L, Lozier J, Lan Y, Maguire M (2001). Defects in development of the kidney, heart and eye vasculature in mice homozygous for a hypomorphic Notch2 mutation. Development.

[b17] Meliss RR, Pethig K, Steinhoff G, Harringer W, Heublein B, Choritz H (1999). Platelet-derived growth factor rather than basic fibroblast growth factor and vascular endothelial cell growth factor is involved in adventitial narrowing causing vascular stenosis in end-stage cardiac allograft vasculopathy. Transplant Proc.

[b18] Neubauer B, Machura K, Schnermann J, Wagner C (2011). Renin expression in large renal vessels during fetal development depends on functional beta1/beta2-adrenergic receptors. Am. J. Physiol. Renal. Physiol.

[b19] Ohlsson R, Falck P, Hellstrom M, Lindahl P, Bostrom H, Franklin G (1999). PDGFB regulates the development of the labyrinthine layer of the mouse fetal placenta. Dev. Biol.

[b20] Pan L, Glenn ST, Jones CA, Gross KW (2005). Activation of the rat renin promoter by HOXD10.PBX1b.PREP1, Ets-1, and the intracellular domain of notch. J. Biol. Chem.

[b21] Reddi V, Zaglul A, Pentz ES, Gomez RA (1998). Renin-expressing cells are associated with branching of the developing kidney vasculature. J. Am. Soc. Nephrol.

[b22] Sauter A, Machura K, Neubauer B, Kurtz A, Wagner C (2008). Development of renin expression in the mouse kidney. Kidney Int.

[b23] Schmahl J, Rizzolo K, Soriano P (2008). The PDGF signaling pathway controls multiple steroid-producing lineages. Genes Dev.

[b24] Senger DR, Davis GE (2011). Angiogenesis. Cold Spring Harb. Perspect. Biol.

[b25] Sequeira Lopez ML, Gomez RA (2011). Development of the renal arterioles. J. Am. Soc. Nephrol.

[b26] Sequeira Lopez ML, Pentz ES, Robert B, Abrahamson DR, Gomez RA (2001). Embryonic origin and lineage of juxtaglomerular cells. Am. J. Physiol. Renal Physiol.

[b27] Sequeira Lopez ML, Pentz ES, Nomasa T, Smithies O, Gomez RA (2004). Renin cells are precursors for multiple cell types that switch to the renin phenotype when homeostasis is threatened. Dev. Cell.

[b28] Soriano P (1994). Abnormal kidney development and hematological disorders in PDGF beta-receptor mutant mice. Genes Dev.

[b29] Stratman AN, Davis GE (2012). Endothelial cell-pericyte interactions stimulate basement membrane matrix assembly: influence on vascular tube remodeling, maturation, and stabilization. Microsc. Microanal.

[b30] von Tell D, Armulik A, Betsholtz C (2006). Pericytes and vascular stability. Exp. Cell Res.

[b31] Wagner C, Jobs A, Schweda F, Kurtz L, Kurt B, Lopez ML (2010). Selective deletion of Connexin 40 in renin-producing cells impairs renal baroreceptor function and is associated with arterial hypertension. Kidney Int.

